# Mitotic and meiotic midbody remnants: Divergent architectures with distinct clinical applications

**DOI:** 10.1016/j.xgen.2026.101386

**Published:** 2026-10-14

**Authors:** Karen Schindler, Ahna R. Skop

**Affiliations:** 1 Department of Genetics, Rutgers, The State University of NJ, Piscataway, NJ 08854, USA; 2 Laboratory of Genetics, UW-Madison, Madison, WI 53706, USA

**Keywords:** midbody, MB, midbody remnant, MBR, meiotic midbody, mMB, meiotic midbody remnant, mMBR, division mode specialization, extracellular vesicles, EVs, localized translation, liquid biopsy, cancer biomarkers, oocyte quality, developmental competence, ARC

## Abstract

Midbody remnants (MBRs), once considered inert cytokinetic debris, are now recognized as architecturally specialized, translationally active organelles whose functions reflect the mode of cell division. This review compares MBR biology in symmetric somatic mitosis and asymmetric oocyte meiosis. In somatic cells, MBRs can be retained or secreted as large extracellular vesicles that carry midbody proteins, RNAs, and translation machinery; their enrichment in stem and cancer cells implicates them in cell-state regulation and identifies them as promising, though unvalidated, substrates for liquid-biopsy biomarkers and therapeutic targeting. In oocytes, meiotic midbodies form a distinctive ring-cap architecture that concentrates local translation and restricts maternal products to the egg, thereby supporting developmental competence. Together, these studies establish division mode as a key determinant of midbody architecture, cargo, fate, and translational potential, linking cytokinesis to cancer biology and reproductive medicine.

## Graphical abstract

**Figure:**
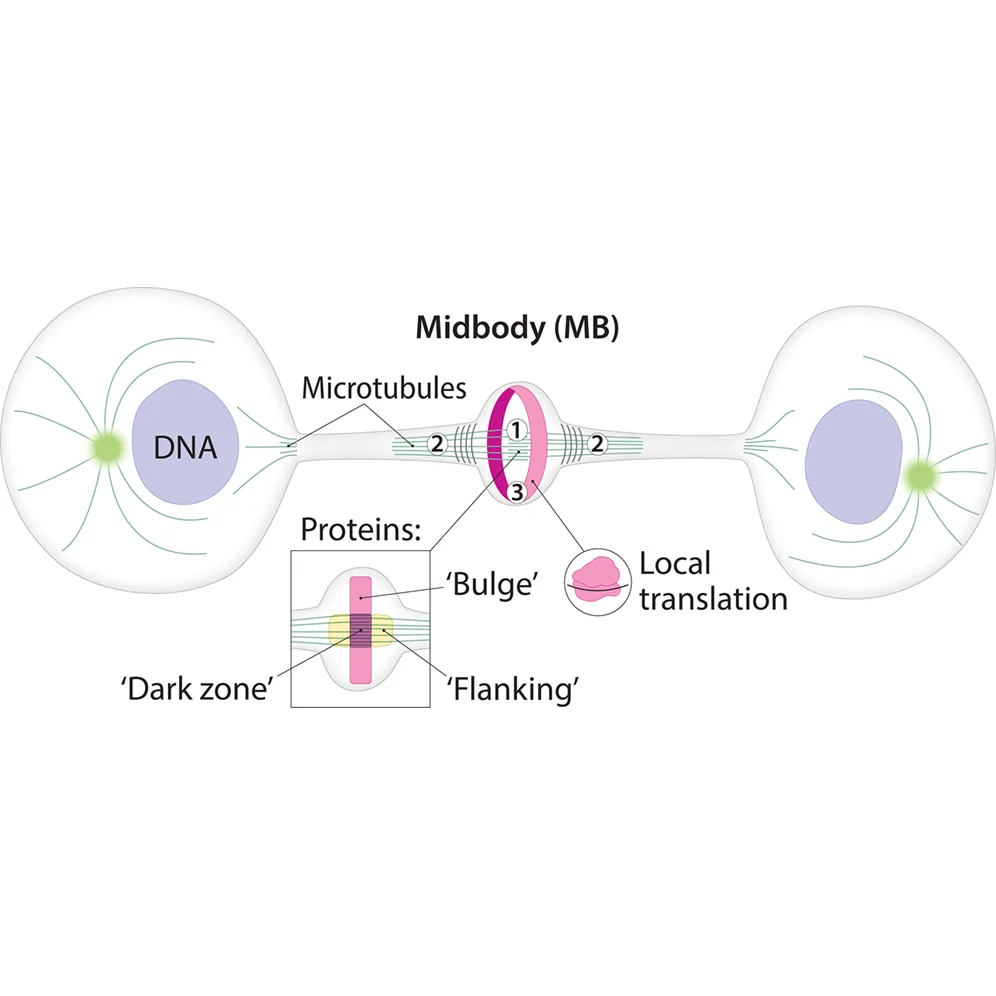

## Teaser

Midbody remnants adopt division-mode-specific functions. Mitotic remnants can act as translating extracellular vesicles with potential relevance to cancer biomarkers, whereas meiotic midbody caps retain maternal products in the egg. Comparing these structures links cytokinesis to cancer diagnostics and reproductive medicine.

## General structure of the midbody

The midbody (MB) is a highly ordered, microtubule-based structure that forms at the center of the intercellular bridge during the final stages of cytokinesis. Architecturally, it comprises three principal domains: (1) the midbody core or “dark zone,” a dense central region enriched with antiparallel microtubule overlaps bundled by the Centralspindlin complex (mitotic kinesin-like protein 1 [MKLP1; encoded by KIF23]; hereafter referred to as MKLP1) and Rac GTPase-activating protein 1 (RACGAP1); (2) the flanking arms, which extend bilaterally toward daughter cells and contain parallel microtubule bundles surrounded by membranes; and (3) the midbody ring/bulge, a cortical scaffold encircling the constriction site, enriched with regulatory proteins such as centrosomal protein 55 (CEP55), citron kinase (CITK), and epithelial cell transforming sequence 2 (ECT2) that coordinate abscission machinery, including the endosomal sorting complexes required for transport III (ESCRT-III) complexes and the severing factor spastin. Functionally, the MB integrates structural, mechanical, and signaling roles—serving as the assembly site for abscission complexes, a local organizer of membrane remodeling, and a transient ribonucleoprotein hub enriched in translation-competent RNA granules. Post-abscission, the MB can be retained intracellularly within one daughter cell or released extracellularly as a membrane-bound midbody remnant (MBR), thereby transitioning from a mechanical scaffold to a signaling organelle that can influence intercellular communication, fate specification, and disease progression.^1^^,^^2^^,^^3^^,^^4^^,^^5^^,^^6^ These three structural domains and their relationship to post-abscission MBR fate are summarized schematically in Figures 1A and 1B.

**Figure 1:**
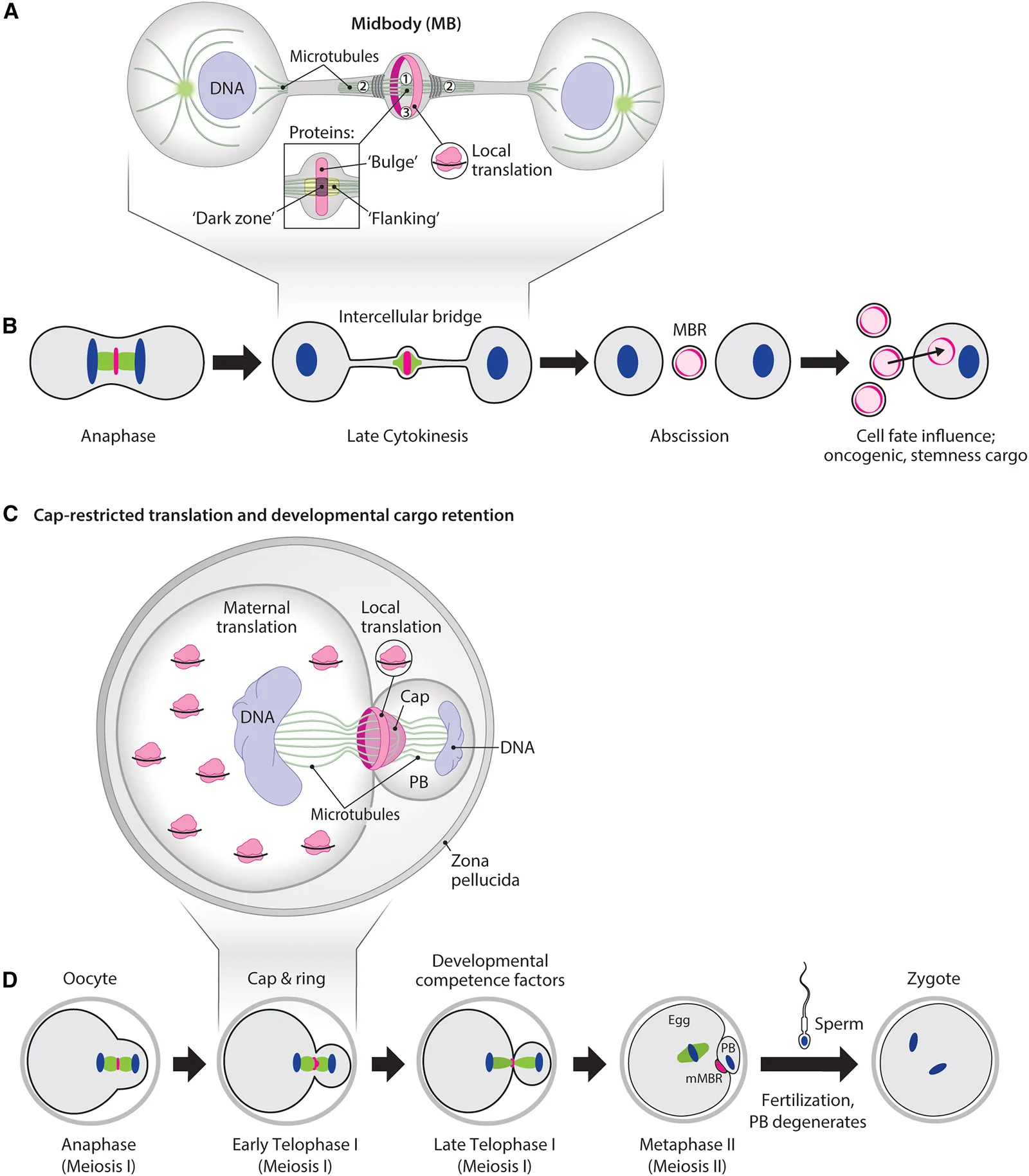
Architecture and functional specialization of mitotic and meiotic midbodies (A and B) The midbody (MB) is a highly ordered microtubule- and membrane-rich structure that forms within the intercellular bridge during late cytokinesis. It contains three principal domains: (1) a central core, or dark zone, composed of antiparallel microtubule overlaps organized by the Centralspindlin complex, including mitotic kinesin-like protein 1 (MKLP1; encoded by *KIF23*) and Rac GTPase-activating protein 1 (RACGAP1); (2) flanking arms containing parallel microtubule bundles that extend toward each daughter cell; and (3) a cortical midbody ring enriched in centrosomal protein 55 (CEP55), citron kinase (CITK), and epithelial cell transforming sequence 2 (ECT2). The MB recruits endosomal sorting complex required for transport (ESCRT) machinery and the microtubule-severing protein spastin to coordinate abscission. It also functions as a ribonucleoprotein (RNP)-rich site of localized translation. Following abscission, the MB may be retained by a daughter cell, degraded, internalized, or released as a membrane-bound midbody remnant (MBR), a large extracellular vesicle (EV) capable of carrying RNA, proteins, and translation machinery. (C) During highly asymmetric oocyte meiosis, the meiotic midbody (mMB) forms between the egg and the first polar body (PB1). The mMB retains a canonical ring but also develops a meiosis-specific cap enriched in Centralspindlin components, including MKLP1 and RACGAP1, that protrudes toward the PB1. This ring-plus-cap organization is associated with asymmetric ball-and-socket microtubule geometry at the egg-polar body interface. Ribosomes, polyadenylated messenger RNAs (mRNAs), and nascent translation products concentrate within the mMB region. The cap acts as a spatial barrier that retains newly synthesized maternal proteins in the egg while limiting their diffusion into the PB1, thereby supporting oocyte developmental competence. (D) After completion of meiosis I, a meiotic midbody remnant (mMBR) remains cortex-proximal between the metaphase II-arrested egg and PB1, within the zona pellucida. Unlike many mitotic MBRs, the mMBR is not freely released into the extracellular environment. Its ultimate fate after fertilization—and whether it is internalized by the zygote or a specific early blastomere—remains unresolved.

## Introduction: Cell division mode diversity in midbody biology

Cell division in animals proceeds through a series of highly coordinated stages that partition chromosomes and cytoplasm into daughter cells. During mitosis, duplicated chromosomes align on the metaphase plate and segregate toward opposite poles, while the actomyosin contractile ring constricts the plasma membrane to form a narrow intercellular bridge between nascent daughters.^5^ A structurally analogous but more asymmetric sequence of events occurs during oocyte meiosis, where reductive divisions generate a large egg and diminutive polar bodies.^7^ In both contexts, formation of an intercellular bridge is followed by cytokinetic abscission, the terminal membrane scission event that physically separates cells and is orchestrated by a specialized cytokinetic organelle—the “midbody (MB).”^5^

The MB is a specialized ribonucleoprotein (RNP)- and microtubule-rich organelle that assembles within the intercellular bridge during early and late telophase, as overlapping spindle midzone microtubules are bundled and compacted by cleavage furrow ingression to complete abscission. Once regarded as a short-lived mechanical scaffold that simply ensures physical separation of daughter cells, the MB is now recognized as a versatile signaling platform whose fate after division profoundly influences cellular physiology. Following abscission, the MB can either be retained within one daughter cell or be released into the extracellular milieu as a membrane-bound “midbody remnant (MBR).” These released MBRs, now classified as large translating extracellular vesicles, are enriched in RNAs, ribosomal components, transcriptional regulators, and epigenetic modifiers, making them potent mediators of intercellular communication and key determinants of cell fate, proliferation, and stemness.^5^^,^^6^^,^^8^^,^^9^

To date, the majority of studies defining MB composition and function derive from mitotic (somatic) divisions, whereas the structure and fate of meiotic midbodies remain comparatively unexplored.^5^ Recent evidence, however, shows that mouse oocytes generate meiotic midbodies (mMBs) with unique architectural and functional specializations. Notably, mMBs possess an asymmetric “cap” enriched in Centralspindlin complex proteins. This cap forms a barrier at the oocyte-polar body interface, retaining nascent translation products within the egg cytoplasm while preventing their loss into polar bodies, and disruption of this cap compromises developmental competence, underscoring its essential role in safeguarding maternal translational products required for zygotic development^7^ (Figures 1C and 1D).

These findings highlight an unappreciated cell-division-mode-specific diversity in MB biology. Mitotic MBRs often function as extracellular conveyors of oncogenic or pluripotency-associated cargo, modulating transformation and tumor progression,^5^^,^^6^^,^^9^^,^^10^ whereas meiotic midbodies (mMBs) and their mMBRs preserve translational and developmental integrity during gametogenesis and early embryogenesis.^7^^,^^11^ This dichotomy underscores how the mode of division—symmetric versus asymmetric, proliferative versus reductive—shapes not only MB architecture but also its biological outcomes. In this review, we therefore compare mitotic and meiotic MB-derived structures across three levels—mechanism, cargo and localized translation, and tissue-level function—to establish a unified framework that links cytokinesis to cancer diagnostics and reproductive medicine, while emphasizing current gaps in large-animal models and clinical validation of MB- and MBR-based biomarkers. A glossary of terms can be found in Box 1.

Box 1 **Glossary**

**Midbody (MB)**: a highly ordered, microtubule-rich structure that forms within the intercellular bridge during the final stages of cytokinesis and coordinates abscission, membrane remodeling, and localized signaling.

**Midbody remnant (MBR)**: the membrane-bound post-abscission structure derived from the midbody; it can be retained within one daughter cell or released extracellularly as a large extracellular-vesicle-like particle that carries RNA, protein, and translational machinery.

**Meiotic midbody (mMB)/meiotic midbody remnant (mMBR)**: midbody-derived structures formed during oocyte meiosis that are specialized for highly asymmetric division, maternal factor retention, localized translation, and developmental competence.

**Polar body (PB)**: a small cell produced during oocyte meiosis that contains chromosomes but relatively little cytoplasm and typically degenerates rather than contributing to the embryo.

**Intercellular bridge/ring canal**: a persistent cytoplasmic connection between daughter or sibling cells that contains midbody-derived material and can permit exchange of cytoplasm, RNAs, proteins, and signals.

**Centralspindlin**: a highly conserved heterotetrameric motor protein complex composed of MKLP1/KIF23 and RACGAP1 that bundles antiparallel midzone microtubules and organizes the central midbody during cytokinesis.

**ESCRT complexes**: a family of membrane-remodeling complexes that drive constriction and membrane scission during cytokinetic abscission, in addition to roles in endosomal sorting and viral budding.

**Maternal mRNAs**: transcripts stored in the oocyte that support meiotic maturation and direct early embryonic development before activation of the zygotic genome.

**Fusion gene**: a gene generated by joining segments of two different genes, usually through chromosomal rearrangement; fusion genes and their transcripts are widely used as cancer biomarkers.

**Liquid biopsy**: a minimally invasive diagnostic approach that detects tumor-derived material, such as DNA, RNA, cells, or vesicles, in blood or other body fluids instead of requiring a tissue biopsy.

**Large extracellular vesicle (large EV)**: a relatively large membrane-bound extracellular particle; in this context, released midbody remnants are considered a distinct cytokinesis-derived large EV population with cargo and protein markers that differ from canonical exosomes and microparticles.

**Developmental competence**: the ability of an oocyte or embryo to support normal fertilization, cleavage, and progression through early development.

## Division-mode-specific mechanisms

### Somatic mitotic pathways

In somatic cells, the canonical mitotic MB coordinates abscission through a hierarchically organized recruitment cascade that couples microtubule architecture to membrane remodeling. Centralspindlin, a highly conserved heterotetrameric motor protein complex comprising the kinesin MKLP1 and RACGAP1, bundles antiparallel microtubules at the spindle midzone and establishes the central MB core, positioning the abscission site within the intercellular bridge. Once furrow ingression has formed a narrow bridge, Centralspindlin recruits the adaptor CEP55 to the MB ring, which in turn engages ESCRT-I and ESCRT-associated factors such as tumor susceptibility gene 101 (TSG101) and ALG-2-interacting protein X (ALIX). These components nucleate ESCRT-III filament assembly along the adjoining bridge arms, where helical ESCRT-III polymers constrict the membrane while simultaneously recruiting the microtubule-severing ATPase spastin and other microtubule-interacting and trafficking (MIT) domain proteins to clear residual spindle microtubules and enable final scission.^4^^,^^5^^,^^12^^,^^13^^,^^14^^,^^15^^,^^16^^,^^17^^,^^18^^,^^19^

Beyond its role as a mechanical scaffold, the MB core harbors a dense ribonucleoprotein matrix that functions as a specialized RNA and translation hub (Table 1). This “midbody granule” or matrix region is enriched for mRNAs encoding transcription factors and regulators linked to fate transitions, pluripotency, and oncogenic transformation, including activator protein 1 (AP-1) components (JUN and FOS) and factors such as Krüppel-like factor 4 (KLF4). The matrix exhibits properties consistent with a phase-separated condensate and supports temporally restricted translation that initiates around the metaphase-to-G1 transition when global protein synthesis resumes. MKLP1 and the retrovirus-derived RNA-binding protein activity-regulated cytoskeleton-associated (ARC) are required to localize and maintain translation-competent RNAs within this compartment, providing concrete examples of proteins that enforce the temporally restricted translation wave at the midbody, while ESCRT-III activity modulates translation levels during the progression to abscission. Although these studies identify key regulators, the broader rules governing selective enrichment of transcription factors and oncogenic transcripts in the midbody granule remain incompletely understood.^9^

**Table 1:** Division-mode-dependent specialization of midbody-derived structures in somatic mitosis, oocyte meiosis, and early embryonic/embryonic stem cell divisions

Feature	Mitotic (somatic)	Meiotic (oocyte)	Early embryo/embryonic stem cells
Architecture	symmetric, short-lived bridge and classic midbody	asymmetric ring-plus-cap midbody between egg and polar body	long-lived bridges and midbodies linking sisters
Translation	local translation at midbody/remnant	cap-restricted translation on egg side	bridge-associated translation between sisters
RNA cargo	RNAs for cell cycle, signaling, stemness, oncogenesis	maternal RNAs for meiosis and early development	RNAs for pluripotency, fate, survival
Fate	remnants inherited, degraded, or secreted as EV-like particles	egg retains key factors; polar body usually degenerates	bridges/midbodies mediate sister-cell communication
Clinical/translational use	cancer biomarkers and therapeutic targets/carriers	markers of oocyte quality and IVF potential	indicators of embryo viability and stem cell health
Key distinction	secreted, signaling midbody EVs; MKLP1/KIF23-centered	MKLP1/KIF23-rich cap essential for egg competence	kinesin family member 2A (KIF2A)-stabilized, long bridges supporting pluripotency

This table compares the architecture, molecular composition, localized translation, intercellular continuity, post-abscission fate, and biological functions of midbody-derived structures across three division contexts. In somatic mitosis, midbodies (MBs) can be inherited, degraded, or released as midbody remnants (MBRs), a distinct class of large extracellular vesicles (EVs) enriched in cytokinetic proteins and translation-associated cargo. In oocyte meiosis, meiotic midbodies (mMBs) and meiotic midbody remnants (mMBRs) acquire an asymmetric ring-plus-cap architecture that spatially restricts maternal RNA and protein retention to the egg. In early embryos and embryonic stem (ES) cells, persistent intercellular bridges containing MB-derived structures can coordinate communication, developmental timing, and cell-fate transitions among sister cells. “Early embryo/ES cell” refers to settings in which canonical MB-derived bridges persist after cytokinesis; a distinct molecularly defined embryonic midbody class has not yet been established.

After abscission, mitotic MBRs can be either retained intracellularly or released as large extracellular vesicles (EVs) that preserve translational activity and carry defined RNA and protein cargos (Figure 1B; Table 1).^8^^,^^9^ Cancer cells, in particular, show pronounced accumulation of MBRs, reflecting both increased production due to high proliferative rates and impaired clearance via autophagy pathways (Figure 2A).^20^^,^^21^ Autophagic degradation of MBRs normally proceeds through recognition of the midbody protein CEP55 by the selective autophagy receptor neighbor of BRCA1 (breast cancer 1) gene 1 (NBR1), but many tumor cells attenuate this pathway, favoring intracellular MBR retention.^21^^,^^22^ Cells enriched for retained MBRs exhibit enhanced anchorage-independent growth and increased tumorigenic potential, and cancer-stem-like subpopulations can show several-fold higher MBR accumulation than bulk tumor cells.^21^

**Figure 2:**
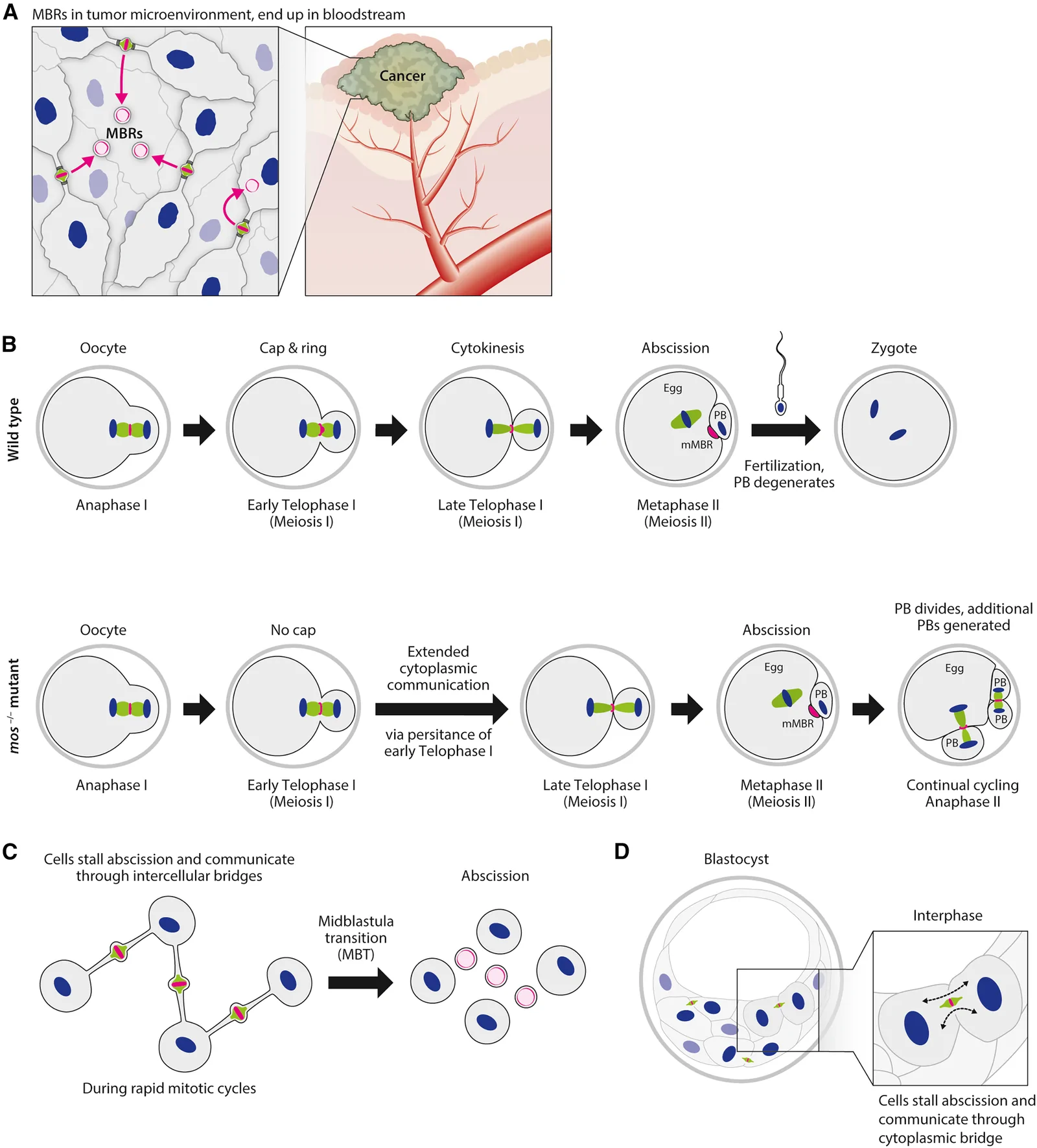
Division-mode-dependent functions of midbody-derived structures in cancer, oocyte meiosis, and embryogenesis (A) In proliferating tumor cells, mitotic MBRs can accumulate intracellularly, be internalized by neighboring cells, or be released as large extracellular vesicles (EVs) into the tumor microenvironment and circulation. MBRs are enriched in cytokinetic proteins, including mitotic kinesin-like protein 1 (MKLP1; encoded by KIF23), and contain RNA and translation-associated cargo that can reflect the proliferative and molecular state of the parental cell. Their abundance and cargo composition create opportunities for MBR-based liquid-biopsy approaches to cancer detection, prognostication, and treatment monitoring. Retained or internalized MBRs may also promote stem-like properties and tumorigenic behavior. (B) In wild-type mouse oocytes, the meiotic midbody (mMB) forms a ring-plus-cap structure between the egg and first polar body (PB1) during highly asymmetric meiosis. The cap is enriched in Centralspindlin components and aligns with asymmetric ball-and-socket microtubule geometry. The mMB acts as a localized translation hub and diffusion barrier that retains newly synthesized maternal products in the egg. In Mos-deficient oocytes, defective mMB-cap assembly and delayed telophase I prolong cytoplasmic continuity between the egg and PB1, permitting aberrant exchange of material into the polar body. This disrupted egg-polar body compartmentalization allows both eggs and polar bodies to continue dividing in the absence of fertilization. (C) During rapid cleavage divisions in early zebrafish embryos, cytokinetic abscission is delayed before the midblastula transition (MBT), generating clusters of blastomeres connected by multiple intercellular bridges containing midbody-like structures. As embryos approach the MBT, increased expression of abscission regulators, including charged multivesicular body protein 4B (CHMP4B), promotes bridge resolution. This developmental transition coincides with zygotic transcriptional activation and the onset of more complex morphogenesis. (D) In mammalian preimplantation embryos, post-cytokinetic MBR release may be delayed, allowing sibling blastomeres to remain connected by persistent intercellular bridges containing MB-derived structures. These bridges permit cytoplasmic exchange and may coordinate cell-cycle progression, transcriptional activation, and cell-fate decisions among connected cells. Regulated bridge persistence and resolution may therefore contribute to embryo geometry, lineage allocation, and developmental competence.

Extracellular MBRs act as horizontally transferable signaling organelles. When taken up by naive recipient cells,^6^^,^^10^ they may deliver oncogenic and pluripotency-associated factors that promote proliferation, survival, and phenotypic reprogramming.^8^^,^^9^^,^^23^ For example, MBRs derived from cancer cells can transform normal fibroblasts toward more cancer-like behaviors, with tissue transglutaminase and cross-linked fibronectin identified as key mediators of this oncogenic transfer. These observations position mitotic MBRs at the interface of cytokinesis, EV biology, and tumor evolution, with their accumulation and molecular composition emerging as promising—but as yet not clinically validated—substrates for liquid biopsy and potential therapeutic targets in oncology (Figure 2A).^5^^,^^24^

Below, we highlight how MBR cargo and translational activity differ from canonical cancer-derived exosomes and microvesicles and why these distinctions may confer unique diagnostic advantages. Together, these differences have direct biomarker implications. Comparative proteomic and transcriptomic studies demonstrate that shed MBRs are enriched for midbody scaffold and abscission regulators (e.g., MKLP1/KIF23, CEP55, and ESCRT components), mitotic kinases, and ribosome- and translation-associated factors,^3^^,^^25^^,^^26^^,^^27^^,^^28^ whereas exosomes and smaller EVs preferentially carry endosome-derived cargos, tetraspanins, and broadly distributed signaling molecules.^29^ In colorectal cancer models, MBRs harbor fusion transcripts and oncogenic signaling networks that are underrepresented or absent in matched exosome preparations,^5^^,^^30^^,^^31^ underscoring that MBRs constitute a distinct cytokinesis-derived large EV population rather than a subset of conventional EVs.^31^

From a diagnostic standpoint, MBR-focused assays may therefore offer several advantages over standard EV biomarkers. First, the physical size and unique marker repertoire of MBRs (large vesicles positive for midbody proteins such as MKLP1 and RACGAP1) enable size- and immunophenotype-based enrichment strategies that can selectively isolate cytokinesis-linked vesicles from the broader EV background,^31^^,^^32^ potentially improving signal-to-noise for fusion gene and mitotic transcript detection in blood or other biofluids. Second, because MBR production scales with proliferative activity and their cargo reflects local translational and cell-cycle states, circulating MBRs could provide dynamic readouts of tumor growth, mitotic stress, and therapeutic response that complement genomically defined, relatively static exosomal markers. Finally, the translationally active nature of MBRs^3^^,^^9^^,^^27^^,^^31^ raises the possibility of monitoring not only transcript presence but also ongoing protein synthesis in tumor-derived vesicles—a dimension of EV-based liquid biopsy that is largely inaccessible when focusing on exosomes or microvesicles alone. In this division-mode-aware framework, MBRs represent a proliferative, cytokinesis-encoded EV class whose biomarker potential arises precisely from their midbody origin and translational competency, rather than from generic vesicle release.

### Meiotic specializations

Prior to entering meiosis, primordial germ cells undergo mitotic amplification in cysts, relying on persistent intercellular bridges to allow sharing of materials proposed to aid in germ cell development.^33^^,^^34^^,^^35^^,^^36^^,^^37^^,^^38^ Upon completion of meiosis I and II, mammalian oocytes execute highly asymmetric meiotic divisions that produce a large egg and diminutive polar bodies, and their mMBs exhibit structural and functional specializations tailored to this developmental context (Figures 1C and 1D; Table 1). In mouse oocytes, Jung and colleagues identified an mMB with a prominent “cap” substructure, minimally composed of the Centralspindlin components MKLP1 and RACGAP1, that protrudes asymmetrically toward the first polar body during early telophase I and remains stably associated with the mMB far longer than typical mitotic midbodies. This cap forms together with a ball-and-socket-like spindle midzone morphology that reflects unequal microtubule abundance between oocyte and polar body compartments. Moreover, super-resolution imaging shows canonical midbody ring proteins such as CITK, ECT2, and CEP55 are confined to the ring, whereas the cap is a meiosis-specific elaboration highly enriched in Centralspindlin, underscoring its distinct molecular identity.^7^

Unlike the persistent intercellular bridges of germ cells in cysts that allow relatively free flow of materials between cells, mMBs function as both translation hubs and diffusion barriers between the egg and polar body (Figures 1C and 1D). Ribosomes, polyadenylated mRNAs, and nascent translation (assayed by homopropargylglycine [HPG] incorporation) concentrate within the mMB region, with the cap demarcating a sharp boundary that restricts newly synthesized products to the egg compartment and prevents their escape into the polar body. Chemical perturbation or laser ablation of the cap disrupts this organization and causes nascent peptides (detected by HPG signal) to leak into the polar body, demonstrating that the cap is essential for maintaining a unidirectional barrier that retains maternal proteins in the egg. Lacefield and colleagues further showed that loss of Moloney murine sarcoma viral oncogene homolog (MOS), the upstream activator of the mitogen-activated protein kinase (MAPK) cascade, phenocopies key aspects of cap disruption: *mos*^−/−^ oocytes exhibit defective mMB caps, a prolonged telophase I, and an extended open intercellular bridge between the egg and first polar body in which fluorescent dextran and other cytoplasmic components can pass into the polar body well into late telophase I, a stage when wild-type oocytes have already sealed the bridge (Figure 2B). In *mos*^−/−^ eggs, this extended connection allows the polar body to assemble bipolar spindles, undergo chromosome segregation with timing similar to anaphase II in the egg, initiate deoxyribonucleic acid (DNA) replication, and even undergo additional divisions after separation from the egg, implicating defective mMB-mediated compartmentalization as a potential contributor to ovarian germ cell tumor formation.^7^^,^^11^

Meiotic MB specializations are not confined to mice. In ascidian oocytes, the mMBR formed after first polar body extrusion (a PB1 meiotic MBR marked by polo-like kinase 1 [PLK1] remains anchored at the cortex and guides the precise spatial positioning of second polar body emission during meiosis II, yielding “tethered polar bodies” in which PB1 and PB2 remain connected via the intervening mMBR. Related tethering phenomena have been documented across diverse invertebrate taxa, indicating that mMBs and their remnants can function as spatial organizers for successive unequal divisions in oogenesis.^39^

Beyond meiosis, early vertebrate embryos maintain persistent intercellular bridges containing embryonic MB-derived structures that span multiple cell cycles. In zebrafish, cytokinetic abscission is delayed during rapid cleavage divisions, so that sibling blastomeres remain interconnected by bridges until the midblastula transition, when abscission initiates as cell cycles lengthen, and zygotic transcription begins (Figure 2C). These long-lived bridges permit exchange of cytoplasmic material and correlate with coordinated transcriptional and morphological behaviors among connected cells, suggesting that embryonic MB-containing bridges contribute to lineage synchronization and patterning during early development (Table 1). Although we refer to “embryonic midbodies” for convenience in Table 1, distinct embryonic MBs have not yet been structurally or molecularly characterized as a separate class; current evidence points to persistence and reuse of canonical mitotic MB-derived bridges in this context.^40^

Advanced reproductive age could add an additional regulatory layer to mMB function. Females are born with their complement of oocytes that are used decades later in reproductive years. Aged mouse oocytes show reduced abundance of translational regulators such as cytoplasmic polyadenylation element-binding protein 1 (CPEB1), premature cyclin B1 expression, accelerated meiotic resumption, and increased spindle instability, and these phenotypes can be phenocopied by partial *Cpeb1* loss in young oocytes.^41^ Whether such age-associated translational defects directly compromise mMB integrity, cap formation, or maternal factor retention—and thereby contribute to declining oocyte quality and fertility—remains an important open question that links MB biology to ovarian aging and reproductive medicine.

## Cargo specialization and intercellular transfer

### Cell-division-mode-specific molecular signatures

Recent transcriptomic and proteomic studies reveal that MBRs derived from mitotic versus meiotic divisions carry distinct, division-mode-specific cargo profiles that mirror their divergent cellular roles. In somatic mitoses, quantitative RNA sequencing (RNA-seq) of purified midbodies identifies a discrete set of roughly two dozen transcripts significantly enriched in mitotic MBs compared with interphase or metaphase microtubule fractions.^7^^,^^9^^,^^27^ These RNAs encode regulators of cell fate, oncogenesis, and pluripotency—such as transcription factors Jun proto-oncogene (JUN), Fos proto-oncogene (FOS), and KLF4—that have no canonical role in cytokinesis yet are central to proliferative and stemness programs, underscoring the MB’s function as a signaling organelle rather than a mere mechanical scaffold. Mitotic MBs and their released MBRs also show enrichment of mitochondrial RNAs and, in colorectal cancer models, carry elevated levels of fusion gene transcripts, including pathogenic MutS homolog 2 (MSH2) rearrangements, positioning MBR cargo as a rich source of candidate liquid biopsy biomarkers that report on both proliferative status and genome instability.^8^^,^^42^^,^^43^^,^^44^ Comparisons between MBRs derived from primary versus metastatic colorectal cancer cells further demonstrate that MBR transcriptomes mirror parental cell identity, with primary-tumor-derived MBRs enriched for epidermal growth factor receptor (EGFR) transcripts and metastatic lines preferentially loading MET proto-oncogene, receptor tyrosine kinase (MET), or metastasis associated in colon cancer 1 (MACC1) fusion species, suggesting that MBR signatures could stratify disease stage and metastatic potential.^42^^,^^43^ However, the molecular rules that govern selective enrichment of these transcriptional and oncogenic factors in mitotic MBs and MBRs, and the sensitivity and specificity of such signatures in large, independent patient cohorts, remain incompletely defined, emphasizing that MBR-based biomarker development is still in an early, exploratory phase.^10^^,^^27^^,^^43^^,^^44^

By contrast, mMBs formed during oocyte maturation are specialized to preserve developmental competence rather than to disseminate signals to neighboring cells. Mouse oocyte mMBs selectively concentrate maternal mRNAs encoding proteins required for fertilization, cell-cycle progression, and early embryogenesis, thereby preserving a local reservoir of factors that support development prior to zygotic genome activation. The asymmetric cap-like structure of the mMB, enriched in Centralspindlin components MKLP1 and RACGAP1, consistently orients toward the polar body and acts as a physical and functional barrier at the oocyte-polar body interface. Unlike mitotic MBRs, which are often released as free EVs, the mMB cap prevents nascent translation products from escaping into the polar body, ensuring that newly synthesized proteins remain within the egg cytoplasm (Figure 1D). Laser ablation of this cap disperses local translation signals and permits leakage of nascent proteins into polar bodies, leading to dramatic reductions in parthenogenetic development beyond the one-cell stage. Together, these findings highlight how division mode dictates midbody cargo composition and fate: somatic MBRs encode export-ready oncogenic and stemness programs with emerging translational potential for cancer diagnostics, whereas meiotic mMBs stockpile and retain maternal effect factors crucial for successful embryogenesis.^7^^,^^11^

### Translational regulation

Midbody-linked structures serve as active sites of localized protein synthesis, with translational dynamics tightly coupled to cell-cycle stage and division mode (Table 2). In somatic mitosis, MB-associated translation is minimal during late telophase, when chromosomes remain condensed and nuclear envelopes are not fully reformed, but becomes robust as cells transition into G1, coincident with chromatin decondensation and global resumption of protein synthesis. Puromycin-based surface sensing of translation (SUnSET) assays reveal that this burst of translation initiates at the midbody and continues after abscission, so that released MBRs function as autonomously translating extracellular vesicles capable of synthesizing proteins at or near the cell surface. Consistent with this finding, MBs and MBRs contain both small and large ribosomal subunits, translation factors, and mRNAs encoding regulators of ribosome biogenesis, collectively establishing a self-contained molecular apparatus for local protein production and providing direct evidence that released mitotic MBRs are translationally active organelles rather than merely RNA-loaded vesicles.^3^^,^^5^^,^^7^^,^^9^^,^^27^

**Table 2:** Mitotic and meiotic midbodies as division-mode-specific signaling organelles

Feature	Mitotic midbody	Meiotic midbody (oocyte)
Division context	symmetric cytokinesis in somatic cells producing two similar daughters	highly asymmetric meiosis producing a large egg and small polar bodies
Overall architecture	canonical core-arms-ring structure at the intercellular bridge	conserved core and arms but modified ring region into a ring plus a prominent cap
Core region	Dense antiparallel microtubule bundle with central “dark zone.”	similar dark-zone core with protein regulator of cytokinesis 1 (PRC1)-enriched discs flanking the central microtubule-free zone
Arms	microtubule arms extending from the core into each daughter cell	microtubule arms extend into the egg and polar body, but are organized asymmetrically
Ring region	single cortical ring containing MKLP1/KIF23 and associated cytokinesis regulators	MKLP1 ring plus a cap-like substructure that bulges toward the polar body
Cap structure	not present; no polarized protruding cap is described in canonical mitotic MBs	distinct cap on egg side that protrudes toward the polar body and is enriched in Centralspindlin components
Microtubule geometry at midzone	generally symmetric overlap between daughter sides	ball-socket asymmetry: ball-like MT termination on egg side, socket-like on polar body side, aligned with cap direction
Dependence on division symmetry	geometry not tightly coupled to daughter cell size inequality	cap formation and ball–socket MT morphology require an asymmetric division; forced symmetry disrupts them.
Assembly dynamics	forms in late anaphase/telophase and persists through abscission; becomes enclosed in a remnant after scission	forms in telophase I and II; cap fully assembled in early telophase I, regresses by late telophase I, with remnant persisting between egg and polar body
RNP/RNA content	functions as an RNA-rich granule with specific mRNAs and RNA-binding proteins	also RNP-enriched with poly(A)+ RNA and RNA-binding proteins localized to the meiotic midbody
Translation machinery	contains both large and small ribosomal subunits and distinct translation initiation factors; supports localized translation around abscission	enriched in small and large ribosomal subunits and active nascent translation localized to the mMB
Spatial control of translation	local translation proposed to influence daughter cell fate and signaling when remnants are inherited	translation spatially restricted to the egg side; the cap defines a boundary that prevents nascent proteins from entering the polar body
Effect of disrupting structure	defects in MB components cause cytokinesis failure, abscission defects, aneuploidy, and altered remnant signaling	disrupting the cap (e.g., via MT perturbation or ablation) causes leakage of nascent proteins into the polar body and reduces egg developmental competence
Abscission timing/checkpoint	abscission coordinated with G1 entry; checkpoints delay scission if midbody or chromatin is abnormal	in normal oocytes, abscission completes by metaphase II; midbody and cap defects prolong telophase I and delay full separation of egg and polar body
Cytoplasmic continuity	intercellular bridge transient; continuity ends with timely abscission	egg and polar body share cytoplasm through an extended bridge; cap integrity helps terminate this connection at the correct time
Remnant fate	midbody remnants can remain attached, be released extracellularly, or be internalized as signaling organelles	meiotic midbody remnants persist between egg and polar body and are hypothesized to act as signaling vesicles or sequestration sites for maternal factors
Roles in fate and disease	remnants influence polarity, stemness, neurite outgrowth, ciliogenesis, and cancer cell behavior	meiotic midbody and its cap support egg developmental competence; defects are linked to abnormal polar body divisions and potential germ cell tumor formation

This table compares the conserved core architecture and context-dependent specialization of midbodies formed during symmetric somatic mitosis and highly asymmetric oocyte meiosis. Both structures contain a microtubule-rich midbody core, flanking arms, a cortical ring, Centralspindlin components, and ribonucleoprotein- and translation-associated machinery that couples local protein synthesis to cytokinetic progression. In somatic cells, post-abscission midbody remnants (MBRs) may be retained, degraded, internalized, or released as large extracellular vesicles (EVs), enabling transfer of cytokinesis-associated RNA and protein cargo between cells. In oocytes, meiotic midbodies (mMBs) acquire a distinctive Centralspindlin-enriched cap and asymmetric ball-and-socket microtubule geometry that concentrate translation at the egg-polar body interface and restrict newly synthesized maternal products to the egg. These contrasts illustrate how division mode determines midbody architecture, cargo deployment, fate, and potential roles in cancer biology, developmental competence, and reproductive medicine.

A key example of functional MB translation is provided by the ESCRT-III subunit charged multivesicular body protein 4B (CHMP4B). Its mRNA is specifically targeted to the midbody through 3′ UTR GA-rich localization elements that are necessary and sufficient for bridge accumulation, and single-molecule RNA FISH detects CHMP4B transcripts in the majority of telophase intercellular bridges. Acute inhibition of translation with puromycin reduces CHMP4B protein levels at the midbody and delays abscission, while washout leads to rapid recovery of CHMP4B at the bridge within minutes, even as cytoplasmic levels remain low, demonstrating that local synthesis rather than diffusion replenishes the midbody pool. ESCRT-III components in turn modulate translation, as their depletion elevates MB-associated protein synthesis, suggesting a feedback circuit in which the abscission machinery tunes local translation to coordinate membrane constriction with protein supply.^3^^,^^27^

Division mode fundamentally reshapes how this translational capacity is deployed. In meiotic divisions, the mMB cap acts simultaneously as a translation hub and a unidirectional diffusion barrier (Figure 1C).^7^ Ribosomes, polyadenylated mRNAs, and nascent translation signals concentrate at the mMB, but newly synthesized proteins are retained on the egg side by the cap and are prevented from diffusing into the polar body, generating a steep asymmetry in protein distribution that safeguards maternal factor enrichment in the oocyte. When mMB caps are malformed, such as in *mos*^−/−^ oocytes, materials freely flow between the egg and polar body, explaining why *mos*^−/−^ polar bodies continue cycling (Figure 2B).^11^ This strategy contrasts sharply with somatic mitotic MBs, in which translated products can either support local abscission events or be packaged into MBRs for release and uptake by neighboring cells. Thus, while both mitotic and meiotic MBs exploit localized translation, mitotic MBRs externalize translationally active cargo to propagate signals between cells, whereas meiotic structures prioritize retention to sustain oocyte quality and developmental competence.

Age and disease further reshape MBR translational competency and cargo, with implications for both aging phenotypes and malignancy. In aging tissues, extracellular vesicles—including those with MB origin—often show altered protein composition, such as reduced apoptotic markers (p53, cleaved PARP, and cleaved caspase-3) alongside increased tetraspanins like CD151, changes that may affect vesicle uptake, signaling potency, and local translation after internalization.^45^ Senescent cells release elevated numbers of EVs carrying proinflammatory and pro-senescence miRNAs that can suppress translational capacity and stress responses in recipient cells, thereby propagating senescence-associated secretory phenotypes across tissues.^46^^,^^47^ In cancer, MBRs are enriched for fusion transcripts, oncogenic mRNAs, and rewired translation regulators that correlate with tumor stage and metastatic behavior,^30^ enabling MBRs to broadcast malignant programs to neighboring cells and possibly precondition distant niches. These state-dependent shifts in MB cargo and translational machinery underscore a broader principle: division mode, physiological age, and disease status converge on MB-linked organelles to sculpt not only the RNA and protein cargo they carry but also when and where that cargo is translated and deployed, creating both vulnerabilities for intervention and opportunities for biomarker discovery.^48^^,^^49^^,^^50^^,^^51^

## Functional specialization in health and disease

### Somatic MBRs: Cancer biology

MBRs follow distinct fates in normal, stem, and cancer cells, with major consequences for cell identity and tumorigenic potential (Skop lab, unpublished data). In most differentiated epithelial cells, post-abscission MBRs are rapidly cleared—primarily via selective autophagy—limiting their persistence and signaling capacity.^21^^,^^52^ In contrast, both stem cells and cancer cells frequently retain MBRs, leading to their accumulation over successive divisions (Figure 2A).^20^ In stem cell systems, MBRs are asymmetrically inherited by the daughter cell carrying the older centrosome, and higher MBR burden correlates with maintenance of an undifferentiated, pluripotent-like state, whereas differentiation is accompanied by a marked reduction in MBR accumulation, consistent with a model in which MBR retention reinforces stemness and MBR release accompanies lineage commitment.^53^^,^^54^^,^^55^^,^^56^

Cancer cells take this logic to an extreme, displaying pronounced MBR accumulation, particularly within tumor-initiating “cancer-stem-cell”-like subpopulations that exhibit enhanced self-renewal and tumor-forming capacity.^20^^,^^21^ Experimental enrichment for MBR-high HeLa or mouse hepatocarcinoma cells yields populations with significantly elevated tumorigenicity compared with MBR-low counterparts from the same line, indicating that MBR burden is not merely correlative but functionally linked to malignant behavior.^2^ Internalization assays further show that cells that engulf post-abscission MBRs upregulate proliferation-associated genes and gain enhanced division rates, survival under anchorage-independent conditions, and increased tumorigenic transformation *in vivo*. When secreted, cancer-derived MBRs are added to quiescent NIH 3T3 mouse fibroblasts, recipient cells internalize these vesicles within hours, then acquire transformed morphology, increased proliferation, and invasive traits, demonstrating that MBRs can act as vehicles of horizontal oncogenic information transfer.^8^

The identification of MBRs as a new biomarker source reframes approaches to monitoring proliferative status and oncogenic programs through non-invasive means. MBRs constitute a distinct class of large extracellular vesicles—typically spanning a several hundred nanometers (800 nm+) up to several microns—that are released into biofluids and can be recovered by standard extracellular vesicle isolation protocols. They are distinguished from other EV subtypes by their cytokinesis-derived biogenesis, enrichment in core midbody proteins such as MKLP1 and CEP55, and, uniquely, by the presence of active translation machinery.^31^ Molecular profiling of MBRs released from colorectal and other cancer cells reveals tumor-specific cargo, including fusion gene transcripts (e.g., MSH2 fusions) and oncogenic signaling components that closely mirror the genetic and transcriptional status of the parental tumor.^8^^,^^23^^,^^30^ Detecting such sequences in circulating MBRs offers a potential route for minimally invasive cancer diagnosis, disease monitoring, and early detection of relapse, although large, prospective clinical studies will be required to rigorously establish sensitivity, specificity, and clinical utility.

Beyond static cargo content, the translational activity of MBRs opens an additional diagnostic window. Because MBRs retain ribosomes and translation factors, their nascent peptide output and associated signatures could, in principle, report on real-time changes in tumor cell state and drug response.^5^^,^^9^ EVs from chemotherapy-exposed cancer cells show altered protein and transporter profiles, including changes in multidrug resistance proteins such as breast cancer resistance protein (BCRP; ABCG2) that correlate with poor therapeutic response,^8^^,^^23^^,^^30^^,^^43^ suggesting that analogous shifts in MBR cargo and translation could act as early indicators of emerging chemoresistance. As proliferating tumor cells continuously generate MBRs while quiescent normal cells do so rarely, circulating MBR abundance may scale with tumor burden and proliferative index, positioning MBRs as both qualitative and quantitative biomarkers in oncology.

Within the broader EV landscape, MBRs occupy a functionally distinct niche. Although canonical tetraspanin markers (CD9, CD63, and CD81) are present on MBs and MBRs, these molecules are shared across exosomes, microvesicles, and other EV classes and thus do not confer specificity. In contrast, the persistence of active translation within MBRs, regardless of isolation method, sets them apart from most exosome and microvesicle populations, which typically lack robust translational capacity. This autonomous translation, combined with enrichment in RNAs encoding pluripotency factors, developmental regulators, and oncogenic drivers, equips MBRs to reprogram recipient cells after uptake. Fibroblasts that internalize cancer-derived MBRs accumulate cytokinetic proteins such as MKLP1 and RACGAP1 along with oncogenic RNAs and subsequently display anchorage-independent growth, enhanced motility, and invasion—hallmarks of malignant transformation. These findings support a model in which MBRs act as mobile, cytokinesis-derived organelles that disseminate and amplify oncogenic programs across a tissue field, promoting metastasis and field cancerization by delivering pro-tumorigenic material to otherwise normal or pre-malignant cells.

### Meiotic MBRs: Reproductive health

mMBRs in oocytes are architecturally and functionally tuned to support reproductive fitness rather than disseminate oncogenic programs. In mouse oocytes, the mMBR is situated between the first polar body and the-metaphase II-arrested egg, and unlike many somatic MBRs, the mMBR is not freely released into the surrounding milieu (Table 2). Instead, it remains nestled at the oocyte cortex within the protective glycoprotein-rich zona pellucida that encases the egg and subsequent preimplantation embryo (Figures 1C and 1D).^7^ This intimate spatial relationship raises the possibility that, after fertilization, the mMBR could be internalized into the zygote or later into a specific blastomere, delivering concentrated maternal RNAs, proteins, or other factors to support early developmental events. Although this has not yet been directly demonstrated, such a mechanism would imply that mMBRs contribute directly to embryonic fitness and might represent an unrecognized axis through which oocyte quality influences outcomes in natural conception and assisted reproductive technologies such as *in vitro* fertilization (IVF).

Early embryogenesis further illustrates how division-mode-tailored MB behavior shapes developmental trajectories. In preimplantation mammalian embryos, classic post-abscission MBR release is delayed, and instead, intercellular bridges containing MB-derived structures persist across multiple cell cycles.^57^^,^^58^ Successful development to the blastocyst stage correlates with precise timing and patterning of the first cleavage divisions, where both the duration of cytokinesis and the synchrony of early mitoses predict embryo viability and implantation potential. Persistent bridges between sibling blastomeres maintain physical connections and permit cytoplasmic exchange, potentially synchronizing cell-cycle progression, transcriptional onset, and fate decisions among connected cells (Figure 2D; Table 1).

Work in zebrafish embryos highlights an extreme version of this strategy. During the rapid, synchronous cleavage divisions preceding the midblastula transition (MBT), cytokinetic abscission is deliberately delayed, generating clusters of blastomeres interconnected by multiple intercellular bridges that share midbody-like structures.^40^ As embryos approach MBT, expression of key abscission regulators such as the ESCRT-III subunit CHMP4B increases, and abscission timing accelerates, breaking these networks into individual cells just as zygotic transcription and more complex morphogenesis begin (Figure 2C). Interconnected sibling cells exhibit more similar transcriptional activation times and morphological behaviors than non-connected neighbors, indicating that shared bridges coordinate developmental timing and may help align lineage trajectories.

In mouse embryos, the relationship between cleavage dynamics, bridge behavior, and patterning is more stochastic but no less critical. Natural variability in the timing of cytokinesis and bridge resolution leads to heterogeneous division schedules among blastomeres, which in turn promote energetically favorable packing configurations and appropriate allocation of cells to outer versus inner positions.^58^ These positional cues feed into trophectoderm versus inner cell mass fate decisions, ultimately shaping blastocyst architecture (Figure 2D). Experimental forcing of tight division synchrony disrupts this balance, resulting in abnormal embryo geometry and perturbed lineage marker expression, underscoring that regulated asynchrony and controlled bridge persistence are integral to robust development.

Taken together, meiotic mMBRs and early embryonic intercellular bridges exemplify a reproductive counterpart to somatic MBR biology. Rather than acting as disseminated oncogenic vectors, meiotic and early embryonic midbody-derived structures safeguard maternal factor retention, coordinate cleavage timing, and help establish correct cell-cell relationships that underpin embryo quality. These functions have direct implications for reproductive medicine: perturbations in mMBR formation, cap integrity, or bridge regulation may contribute to age-related declines in egg quality, aneuploidy, implantation failure, and early pregnancy loss. Conversely, imaging or molecular interrogation of mMBRs and embryonic bridges could provide novel markers of egg and embryo competence, guiding embryo selection and optimization of IVF protocols in the clinic.

## Translational applications and clinical horizons

### Cancer diagnostics and therapeutics

Incorporating MBR analysis into liquid biopsy platforms would create new opportunities for cancer detection and monitoring that complement tissue-based diagnostics. MKLP1, a core cytokinetic motor protein enriched in MBs and MBRs, has emerged as a pan-cancer proliferation and prognostic marker, with elevated MKLP1 expression correlating with higher tumor grade and worse overall survival in malignancies such as lung cancer, pancreatic ductal adenocarcinoma, clear cell renal cell carcinoma, and glioma.^59^^,^^60^^,^^61^^,^^62^^,^^63^ While these insights have largely come from tumor tissue analyses, circulating MBRs could, in principle, provide minimally invasive, longitudinal access to similar biology through serial blood sampling. Liquid biopsy methods already detect oncogenic rearrangements and gene fusions in circulating tumor DNA at tumor fractions above roughly 1%, and early work indicates that MBR-derived RNA offers a complementary substrate enriched for fusion transcripts and cytokinesis-linked cargo.^8^^,^^64^^,^^65^ In colorectal cancer models, transcriptomic profiling of released MBRs reveals MSH2 fusion transcripts and differential loading of EGFR versus MET/MACC1 species between primary and metastatic lines, suggesting that circulating MBR signatures could both diagnose malignancy and report on metastatic status. However, these studies are currently limited to cell line and small patient cohorts, and large, prospective clinical trials have not yet validated MBR-based assays for routine cancer diagnosis or monitoring.^30^

The unique translational activity of released MBRs provides an additional functional readout beyond static nucleic acid or protein measurements. Unlike most conventional EVs, which lack robust autonomous translation, MBRs retain ribosomes and translation factors and continue protein synthesis after release.^9^ This capacity raises the possibility of using MBR “translation signatures” to monitor therapy response in real time. EVs from chemotherapy-treated cancer cells exhibit treatment-induced shifts in protein and transporter composition, including changes in multidrug resistance proteins such as ATP-binding cassette subfamily G member 2 (BCRP), and these alterations can correlate with clinical response or resistance.^66^^,^^67^ Extending this concept, longitudinal analysis of MBR-associated ribosome occupancy, nascent peptide production, or expression of resistance-related factors could provide early warning of adaptive drug resistance—potentially months before radiographic progression—allowing clinicians to adjust regimens proactively.^68^^,^^69^ Because proliferating tumor cells continuously generate MBRs while quiescent normal tissues do so at low levels, circulating MBR burden and activity might also serve as dynamic indicators of tumor proliferative load, although such applications remain to be formally tested in patient cohorts.

Beyond diagnostics, MBRs and related cancer-derived vesicles are being explored as therapeutic tools and targets. Cancer-cell-derived EVs exhibit intrinsic tumor-tropic properties and efficient cellular uptake, which has motivated their use as drug delivery vehicles.^70^ In patients with malignant pleural effusion from advanced lung cancer, intrathoracic administration of cisplatin-loaded A549 human lung adenocarcinoma-cell-derived EVs achieved marked reductions in malignant cells in effusions, illustrating that engineered cargo in tumor-derived vesicles can exert potent anti-tumor effects *in vivo*.^71^ Analogous strategies using MBRs could exploit their natural uptake pathways and midbody-enriched surface markers to deliver chemotherapeutics, RNA therapeutics, or immune modulators directly to proliferating tumor cells or cancer stem cell niches. However, because native-cancer-derived EVs and MBRs can also propagate oncogenic signals and drug resistance, clinical translation will require careful control of cargo, possibly by using MBRs from engineered non-malignant cells, *ex vivo*-loaded MBR-like particles, or vesicles stripped of endogenous oncogenic content. At present, such MBR-based therapeutic approaches are conceptual and preclinical.^72^^,^^73^

MBRs themselves constitute a targetable node in tumor biology. Their accumulation in cancer reflects both increased production and impaired autophagic clearance, and their cargo loading depends on specific RNA-binding and trafficking factors.^21^^,^^52^ The retrovirus-derived protein ARC, for example, is required for assembling translation-competent RNA granules at MBs and for packaging RNA into released MBRs. Perturbing ARC function, Centralspindlin-dependent loading, or other midbody-specific trafficking pathways could blunt horizontal transfer of oncogenic transcripts and signaling complexes, thereby limiting field cancerization and metastatic seeding driven by secreted MBRs.^8^^,^^9^^,^^10^ Combining such strategies with standard cytotoxic or targeted therapies may help prevent the vesicle-mediated spread of malignant traits within and beyond the primary tumor, but these ideas remain to be evaluated *in vivo* and in clinical settings.^70^

### Reproductive medicine

In reproductive medicine, meiotic MB caps and remnants offer a potential new layer of biomarkers and intervention points to improve egg and embryo selection. Current IVF practice relies heavily on morphological criteria—cell number, symmetry, and fragmentation—and time-lapse-derived morphokinetics, supplemented in some settings by preimplantation genetic testing for aneuploidy (PGT-A) or analysis of spent media, yet implantation rates remain suboptimal and morphologic grading alone only imperfectly predicts live birth.^74^^,^^75^ Because mMBRs in eggs are large, cortex-proximal structures situated between the metaphase-II-arrested egg and the first polar body, they could be visualized with high-resolution light microscopy prior to fertilization without additional manipulation. The presence, size, and structural integrity of the mMB cap, together with its local translation activity, could thus serve as non-invasive indicators of oocyte competence and developmental potential, although this possibility has not yet been tested in clinical cohorts. Integrating mMBR imaging with existing artificial intelligence (AI)-enhanced morphokinetic pipelines,^76^ and noninvasive metabolomic or Raman spectroscopic profiling of spent media,^77^ may ultimately yield composite scores with higher predictive power for implantation and ongoing pregnancy than morphology alone, but such multimodal strategies remain a forward-looking goal.

Assessing translational competency offers a mechanistically grounded approach to stratifying egg quality, particularly in the context of advanced maternal age. Age-related fertility decline is closely tied to defects in oocyte proteostasis and translation, including reduced expression or function of regulators such as CPEB1 and eukaryotic translation initiation factor 4E family member 1B (eIF4E1B), impaired spindle assembly, and increased chromosomal segregation errors.^41^ Diagnostic assays that quantify translation capacity—via ribosome profiling, nascent peptide labeling, or measurement of key translation regulators in oocytes or associated cumulus cells—could help identify patients whose oocytes are especially translation-deficient. Those with pronounced deficits might be candidates for tailored stimulation protocols, earlier transition to donor oocytes, or enrollment in trials of translation-supportive interventions, whereas patients with preserved translational capacity despite chronological age could be counseled toward single-embryo transfer and more conservative treatment intensification. Transcriptomic profiling of cumulus cells, which reflect the oocyte microenvironment, is already under investigation as a non-invasive proxy for oocyte quality and could be extended to incorporate markers of mMB formation and translation-linked gene expression.^78^^,^^79^

Therapeutically, modulating mMB-linked pathways and enhancing oocyte translation are attractive concepts but are still at a very early stage. Preclinical and emerging clinical data suggest that metabolic and antioxidant interventions can restore aspects of oocyte competence. Nicotinamide mononucleotide (NMN), a precursor of nicotinamide adenine dinucleotide (NAD^+^), improves mitochondrial function, redox balance, and apoptotic signaling in aged and metabolically stressed oocytes, restoring expression of key developmental regulators and improving blastocyst formation in animal models.^80^ A patented formulation combining NMN with the antioxidant L-ergothioneine (EQOQ) has been reported to enhance oocyte and embryo quality and improve implantation and clinical pregnancy rates in women with diminished ovarian reserve, though larger controlled trials are needed to validate these findings,^81^ and these studies have not directly interrogated mMBR structure or function. Additional supplements such as myo-inositol and melatonin have been associated with improved embryo quality and fertilization rates in specific patient groups, including those with polycystic ovary syndrome.^82^^,^^83^ At present, any connection between these interventions and direct modulation of mMBs or mMBRs remains speculative.

More targeted interventions could eventually aim to directly modulate maternal mRNA translation machinery. eIF4E1B is indispensable for selective translation of key maternal transcripts during oocyte maturation and early embryogenesis, and CPEB1 orchestrates poly(A)-tail-dependent translational activation of many of the same RNAs.^41^ Pharmacological strategies that stabilize or enhance the activity of these factors—or that correct upstream signaling defects that impair their function—could, in principle, help reverse age-related translational decline and restore developmental competence, but no such therapies have yet entered clinical testing. Ultimately, combining mMB-focused diagnostics (cap integrity, localization, and translation activity) with individualized regimens that support oocyte translation and mitochondrial health could form the basis of a precision reproductive medicine framework. In this division-mode-aware view, mitotic MBRs inform cancer diagnostics and therapeutics, while meiotic mMBs and their translational landscape guide interventions to optimize fertility and embryo viability, with both applications still in their early translational phases.

## Future directions

The transformative potential of the MBR as both a fundamental signaling paradigm and a clinical tool hinges on addressing key mechanistic questions and building the infrastructure needed for rigorous translation. Multi-omics integration is a particularly important next frontier, moving beyond single-platform descriptions of MBR cargo toward unified molecular maps that link RNA, protein, and lipid composition to function.^9^^,^^25^^,^^27^^,^^28^^,^^84^ Current studies profiling MBR transcriptomes, midbody proteomes, or vesicle lipidomes each capture only slices of this complexity. Coordinated genomic, transcriptomic, proteomic, and lipidomic analysis of matched midbodies and MBRs will clarify how specific miRNA-mRNA-protein networks regulate local translation, how membrane lipid composition shapes vesicle stability and uptake, and which composite signatures distinguish developmental from oncogenic outcomes. Direct, side-by-side multi-omics comparisons of mitotic versus meiotic MBRs should reveal division-mode-specific cargo-loading rules, identify master regulators controlling translational competency, and define molecular fingerprints predictive of stemness, tumorigenicity, or developmental competence. Single-cell multi-omics combined with vesicle profiling will be especially powerful for tracing the cellular origins of heterogeneous MBR populations and discovering rare disease-associated signatures.

Dissecting translation control, uptake pathways, and regulatory circuits that govern MBR fate will require tightly integrated imaging, biochemical, and genetic approaches. Central questions include how specific mRNAs are targeted to midbodies, how cap-dependent versus cap-independent translation is deployed within MBRs, and which endocytic routes mediate MBR internalization into different recipient cell types. Live-cell and super-resolution imaging of nascent translation, combined with acute perturbations of ESCRT-III, ARC, and Centralspindlin, will clarify how abscission machinery feeds back on local protein synthesis. Structural studies of ARC capsid-like assemblies and their RNA recognition modes can guide rational strategies to modulate cargo loading, while clustered regularly interspaced short palindromic repeats (CRISPR)-based screens probing factors that alter MBR accumulation versus degradation may reveal druggable nodes that tune MBR-mediated signaling in stem and cancer cells.^5^ A major goal for the next 5 years will be to translate these mechanistic insights into testable hypotheses about how selective enrichment of transcription factors and oncogenic transcripts in MBs and MBRs is achieved *in vivo.*

Model organism genetics and cross-species comparative studies will remain essential for defining conserved principles and prioritizing therapeutic targets.^5^ Proteomic work showing that the vast majority of mammalian midbody proteins have *Caenorhabditis elegans* homologs, many required for cytokinesis, underscores the deep evolutionary conservation of core machinery.^26^ Studies in Drosophila, zebrafish, and mouse embryos reveal that midbody-to-ring canal transitions and persistent intercellular bridges are reused in development, with regulators such as citron kinase, known as Sticky in *Drosophila* acting across tissues and species.^85^^,^^86^^,^^87^ The observation that a substantial subset of midbody proteins also functions in neuronal and muscle morphogenesis suggests that cytokinesis modules have been repeatedly co-opted to build specialized intercellular structures. Systematic RNA interference (RNAi) or CRISPR screens in tractable models can rapidly identify factors required for meiotic cap assembly, somatic MBR release, or bridge persistence, while evolutionary genomics can distinguish conserved domains from lineage-specific innovations. To date, midbody and MBR biology has been explored predominantly in cell lines and small animal models; large-animal studies (e.g., dog, pig, and non-human primate) are essentially absent and represent a key translational gap. Cross-species validation that markers such as MKLP1 report proliferative states in both small and large animal models and human tumors will strengthen confidence in their clinical deployment.^63^^,^^88^

Robust clinical translation of MBR-based diagnostics and therapeutics will depend on large-scale biomarker validation, methodological standardization, and coordinated data sharing. Proof-of-concept studies detecting MBR-like vesicles and fusion transcripts in patient biofluids, and multi-cancer EV-based classifiers with high area under the curve (AUC) values for early detection, are promising but must be followed by prospective multi-center trials with sufficiently large and diverse cohorts.^8^^,^^64^^,^^89^^,^^90^^,^^91^ Obstacles familiar from broader extracellular vesicle biomarker development—assay reproducibility, pre-analytical variability, and regulatory qualification—will need to be addressed explicitly for MBR-focused assays, including reproducible size- and marker-based separation of large MBRs from smaller EVs and lipoproteins while preserving labile RNA cargo. Division-mode-specific applications will require tailored workflows: for reproductive medicine, protocols that preserve mMB caps and their translational activity in follicular fluid and culture media; for oncology, pipelines that robustly enrich circulating mitotic MBRs and support fusion gene detection at low abundance.^91^^,^^92^^,^^93^^,^^94^^,^^95^^,^^96^^,^^97^

Collaborative infrastructures modeled on successful early detection and biomarker networks will be pivotal. Multi-institutional consortia that harmonize sample collection, isolation, and analytical pipelines, coupled with centralized or federated multi-omics databases, would enable meta-analyses with the power to detect clinically meaningful effects and validate division-mode-specific markers across populations and disease contexts. Such efforts will be crucial to move MBR biology from conceptual paradigm to everyday clinical practice in both oncology and reproductive medicine, transforming mitotic and mMB remnants from niche organelles into broadly useful diagnostic and therapeutic platforms.^73^^,^^98^

## Conclusions

Midbody structures have moved from being regarded as disposable cytokinetic debris to being recognized as division-mode-specific signaling organelles with direct implications for cell fate, development, and disease. Mitotic MBRs act as released intercellular messengers that transport oncogenic and pluripotency-associated cargo, whereas mMB caps and remnants function as retention barriers that preserve maternal factors essential for developmental competence. These contrasting architectures, cargo repertoires, and translational programs reflect evolutionarily divergent strategies for using cytokinetic structures to regulate fate decisions and intercellular communication (Table 2).^5^

This framework opens dual, but still nascent, translational trajectories. In cancer, circulating MBRs provide a promising liquid biopsy substrate for fusion gene detection and dynamic translational readouts, complementing tissue-based MKLP1 prognostication and suggesting new targets such as ARC-dependent cargo loading or MBR-mediated drug delivery. In reproductive medicine, visualization and functional assessment of mMB caps and remnants could, in principle, enable more accurate oocyte quality evaluation and embryo selection, addressing long-standing limitations of morphology-based criteria, although these applications remain to be validated in clinical studies. Division-mode-specific targeting of midbody-linked pathways—suppressing oncogenic information transfer in somatic contexts while bolstering maternal translation and cap integrity in meiotic settings—exemplifies how organelle-centered biology may ultimately be leveraged for precision medicine across oncology and fertility care.

## Acknowledgments

We thank members of the Schindler and Skop laboratories for insightful discussions and feedback on this manuscript, especially Elizabeth Torr (Skop Lab) for carefully reading the text. We are especially grateful to our colleagues in the cytokinesis, extracellular vesicle, cancer genomics, and reproductive biology communities whose work has laid the foundation for the concepts discussed here. We also thank Adam Steinberg (artforscience.com) for generating the figures for this review. Funding: K.S. is supported by the 10.13039/100000002National Institutes of Health (NIH) through grants R35 GM122475 and R21 HD121782. A.R.S. is supported by 10.13039/100000002NIH R35 GM161113, R21MH138941, and a 10.13039/100007015University of Wisconsin-Madison 10.13039/100001395Wisconsin Alumni Research Foundation (WARF) Accelerator grant (135-AAP5482). The content is solely the responsibility of the authors and does not necessarily represent the official views of the 10.13039/100000002National Institutes of Health, WARF, or other funding agencies.

## Author contributions

Conceptualization, K.S. and A.R.S.; writing – original draft, K.S. and A.R.S.; writing – review and editing, K.S. and A.R.S.; visualization, K.S. and A.R.S.; funding acquisition, K.S. and A.R.S.; supervision, K.S. and A.R.S.

## Declaration of interests

A.R.S. is a co-founder, equity holder, and officer of aMBR Genomics, Inc., a company developing technologies related to MBRs. This financial interest has been disclosed to the University of Wisconsin-Madison and is managed in accordance with institutional policies.

## Declaration of generative AI and AI-assisted technologies in the manuscript preparation process

During the preparation of this work, the authors used Perplexity Pro to assist with refining sentence structure, identifying relevant references, and assessing whether reviewer-requested revisions had been adequately addressed and figures and tables were all referenced in the text. All AI-generated outputs were critically evaluated and edited by the authors as necessary. The authors take full responsibility for the content and integrity of the published article.
